# A Case for Anti‐IgE Vaccination

**DOI:** 10.1111/all.70287

**Published:** 2026-03-11

**Authors:** Paul Engeroff, Zahra Gharailoo, Monique Vogel, Martin F. Bachmann

**Affiliations:** ^1^ Department of BioMedical Research (DBMR) University of Bern Bern Switzerland; ^2^ Department for Rheumatology and Immunology University Hospital of Bern Bern Switzerland

**Keywords:** allergy, Antibodies, asthma, atopy, basophils, CD23, chronic rhinosinusitis, FcεRI, food allergy, mast cells, omalizumab, urticaria

## Abstract

Immunoglobulin E (IgE) plays a central role in allergic diseases by binding to the high‐affinity receptor FcεRI on mast cells and basophils, where allergen‐induced crosslinking triggers potent inflammatory responses. Various mechanisms by which IgE responses are generated and functionally regulated remain elusive despite many years of research. Nevertheless, monoclonal anti‐IgE therapy with omalizumab has transformed allergy treatment and proven to be safe and effective in various allergic indications. A remaining limitation of omalizumab is its high cost and requirement for repeated dosing, which limits accessibility. Vaccination against IgE theoretically offers a promising, cost‐effective alternative, but long‐standing safety concerns have slowed its development. Here, we review emerging concepts in IgE biology and therapeutic IgE neutralization. Recent research demonstrates that vaccine‐induced anti‐IgE antibodies can selectively neutralize free IgE while sparing FcεRI‐bound IgE, thereby avoiding effector cell activation. This mechanism mirrors the behavior of natural anti‐IgE autoantibodies, which may regulate physiological IgE homeostasis. Together, these novel insights indicate that anti‐IgE vaccination is safe, biologically grounded, and a compelling strategy for the long‐term control of IgE‐mediated allergic disease.

## 1 The Biology and Clinical Relevance of IgE

### 1.1 Immunoglobulin E

Immunoglobulin E (IgE) was first identified in 1966 by Kimishige and Teruko Ishizaka [1]. The discovery resolved the long‐standing mystery of “reaginic” antibodies, which were thought to cause allergic responses but had not been classified within the existing immunoglobulin framework. IgE is the least abundant antibody isotype in serum, with concentrations typically in the nanogram per milliliter range. In B cells, IgE production is fundamentally driven by IL‐4 and STAT‐6 signaling and T helper cell co‐stimulation via CD40L [2, 3, 4, 5, 6]. Innate IL‐4 sources (NKT, ILC2s, and basophils) and dendritic cell programmes may further shape the magnitude and quality of IgE responses by providing early polarizing signals [7, 8, 9, 10, 11, 12, 13].

It has now become relatively clear that within secondary lymphoid organs (SLO), T follicular helper cells (Tfh) and regulatory cells (Tfr) are essential in controlling the local IL‐4 signals needed for driving functional IgE responses from B cells [14, 15, 16, 17]. The detailed mechanisms by which Tfh and Tfr are fine‐tuned in SLOs to generate functional IgE responses is the subject of ongoing research. One study has shown that IL‐4^+^IL‐13^+^Tfh are critical cells in generating anaphylactic IgE responses [18]. Another study proposed that TGF‐β signaling may restrain anaphylactic IgE responses from Tfh [19]. Other studies have found that hyperactivation of Tfr cells may contribute to the generation of anaphylactic IgE, possibly by enhancing IgE responses via IL‐4 or via suppression of IgG responses that could protect from anaphylaxis [20, 21, 22].

### 1.2 Remaining Controversies on the Generation of IgE

Several fundamental aspects of IgE biology remain controversial. First, the beneficial evolutionary purpose of IgE and its functions beyond allergy are still debated. Studies have proposed a role in host defense against parasites, venoms, or cancer, although overall, the underlying mechanisms are still insufficiently understood [23, 24, 25, 26, 27, 28, 29, 30, 31, 32]. Thus, the extent to which these proposed protective functions critically depend on IgE, and whether they justify the evolutionary conservation of IgE, remains unresolved.

Another area of controversy concerns the precise mechanisms of in vivo IgE class‐switch recombination (CSR) and affinity maturation. Unlike IgG, which undergoes CSR and somatic hypermutation within germinal centers (GCs), IgE appears to follow atypical pathways. Studies discuss controversially whether IgE CSR occurs “classically” directly from IgM or sequentially from IgG to IgE [2, 4, 33, 34, 35, 36].

IgE^+^ GC B cells are rare and often short‐lived, displaying a transcriptional program biased toward terminal differentiation rather than cycles of proliferation and mutation. Most IgE expressing B cells are short‐lived plasmablasts and the existence of long‐lived plasma cells and IgE memory B cells is an ongoing topic of debate [5, 37, 38]. In contrast to IgG memory compartments, IgE^+^ memory B cells are extremely scarce (or possibly even absent) in both mice and humans. Recently, distinct populations of IgG and IgE co‐expressing B cells have been defined as IgE memory B cells “MBC2s,” arguing that IgE memory is contained within the IgG compartment. In turn, others have found that sequential switching via IgG is not required for functional IgE responses, which raises questions on this concept [39, 40, 41, 42].

IgE plasma cells tend to be short‐lived and are generally thought to decline rapidly in the absence of type 2 inflammation, as demonstrated in various atopic patients undergoing dupilumab (anti‐IL‐4R) treatment [5, 43]. However, recent studies in mice have detected persisting IgE plasma cell responses in spleen or bone marrow [44, 45, 46]. Still, whether long‐lived, specific IgE plasma cells exist and whether they are functionally relevant remains controversial [5, 47].

Several recent studies have observed a form of “convergent” IgE evolution where unrelated individuals bind identical epitopes and, more surprisingly, show identical IgE gene rearrangement sequences [37, 48, 49, 50]. Potentially, these findings suggest the existence of a preexisting germline‐encoded IgE repertoire, which could eliminate the need for GC affinity maturation.

**FIGURE 1:**
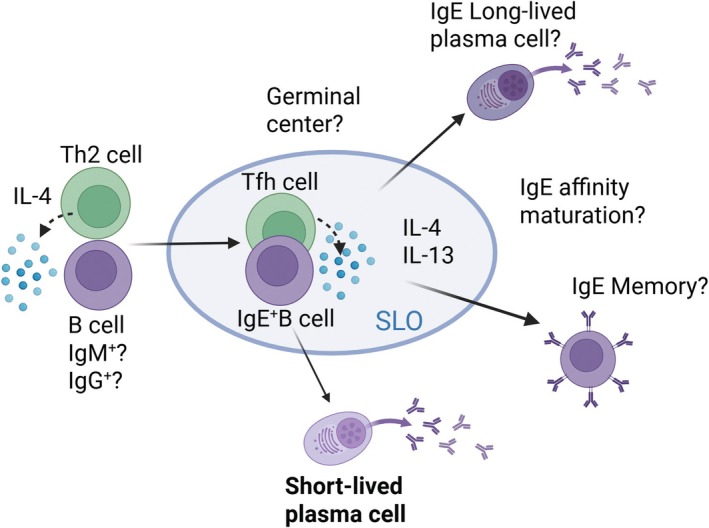
The generation of IgE. Despite years of research, besides a critical need for IL‐4 and T cell help, with essential roles of follicular helper T cells (Tfh), various mechanisms of the antigen‐specific IgE response from secondary lymphoid organs (SLOs), including IgE class‐switching, affinity maturation, the generation of plasma cells and memory B cells remain debated.

Of note, similar germline‐encoded antibody responses have been observed against pathogens and autoantigens [51, 52, 53]. In summary, various aspects of the B cell IgE response still remain somewhat mysterious today (Figure 1).

### 1.3 Structure Function Relationships for IgE

Structurally, IgE comprises two heavy chains and two light chains, with each heavy chain containing four constant domains (Cε1–Cε4) instead of the three found in IgG. The flexibility of its Cε2 domains allows IgE to adopt bent conformations, influencing and tightening receptor engagement [54, 55, 56, 57, 58]. IgE exerts its biological activity through interactions with two main receptors: the high‐affinity receptor FcεRI, expressed on mast cells and basophils, and the low‐affinity receptor CD23 (FcεRII), expressed on B cells and an array of other immune cells [59, 60, 61, 62, 63, 64].

Cross‐linking antigen and IgE‐FcεRI complexes on effector cells triggers immediate degranulation, releasing histamine, leukotrienes, and cytokines that cause allergic symptoms. CD23, in contrast, plays a regulatory role in IgE homeostasis, modulating IgE production and regulating antigen transport and presentation [61, 65, 66, 67, 68, 69, 70, 71, 72].

Binding to FcεRI is essentially irreversible under physiological conditions and the receptor‐IgE complex is not internalized, enabling mast cells and basophils to remain sensitized with surface‐displayed IgE for long periods. IgE has been shown to adopt distinct conformational states in its free form (“closed” conformation) and its FcεRI‐bound form (“open” conformation) [56, 57, 58]. The simultaneous binding of the two IgE receptors may be prevented by this conformational mechanism, as receptor binding locks IgE in its “closed” or “open” conformation [73, 74, 75]. While FcεRI binds free IgE with significantly higher affinity than CD23, IgE in the form of an immune complex preferentially binds to CD23 over FcεRI [76, 77, 78].

**FIGURE 2:**
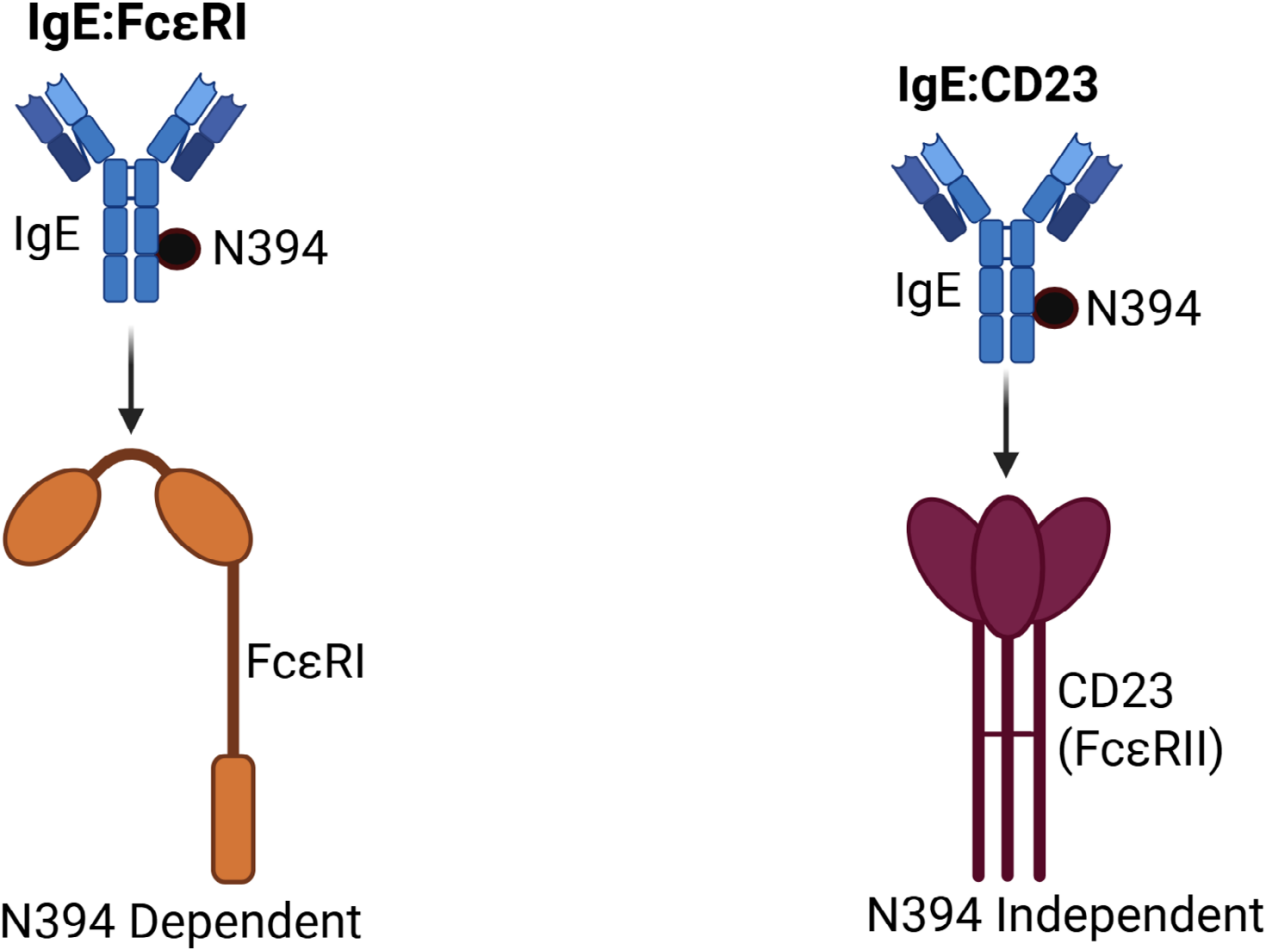
Differential glycan‐dependence of the two IgE receptors FcεRI and CD23. Schematic illustrating the two principal IgE receptors and their distinct requirements for IgE glycosylation. Binding to the high‐affinity receptor FcεRI strictly depends on the highly conserved N‐linked glycan in the IgE Fc region (N394), whereas the low‐affinity receptor CD23 recognizes IgE independently of N394.

IgE is heavily glycosylated, possessing seven N‐linked glycosylation sites, one of which (N394 in humans, N384 in mice) is a highly conserved high‐mannose type glycosylation site critical for binding to its high‐affinity receptor FcεRI. In contrast, the binding of IgE to CD23 occurs independently of N394, even though CD23 is a C‐type lectin that can bind carbohydrates in other contexts [79, 80, 81, 82, 83, 84, 85] (Figure 2).

### 1.4 The Rise of IgE‐Mediated Diseases

IgE is a central effector in type I hypersensitivity conditions. Recent research has suggested that IgE dysregulation may also be involved in driving autoimmune inflammation, although the mechanisms remain largely unclear [86, 87, 88].

Classical IgE‐mediated diseases including allergic asthma, atopic dermatitis, food allergy, allergic rhinitis, and chronic urticaria constitute a major and growing global health burden [89, 90, 91, 92]. The rising prevalence of atopic diseases is theorized to arise from impaired early‐life immune education and modern environmental pressures. Central to this are the hygiene hypothesis, the microbiota dysbiosis hypothesis, and the epithelial barrier hypothesis, which together propose that reduced microbial exposure, altered commensal communities, and weakened skin and mucosal barriers promote IgE‐biased immunity [93, 94, 95].

Large‐scale reviews and meta‐analyses consistently show that 30%–50% of children in industrialized countries exhibit IgE sensitization whereas 20%–30% manifest at least one allergic disease, with prevalence rising during the late 20th century and remaining highest in urban, resource‐rich environments [96, 97, 98].

Thus, IgE‐mediated diseases affect hundreds of millions of people worldwide and are responsible for substantial direct medical costs, driven by recurrent physician visits, emergency care for severe exacerbations or anaphylaxis, lifelong medication use, and costly biologic therapies. Indirect costs are loss of productivity, missed school and work days, sleep disruption, and the long‐term socioeconomic impact of chronic symptoms. In low‐ and middle‐income countries, lack of access to specialist care and expensive treatments further amplifies morbidity and health disparities. Beyond economic consequences, the reduction in quality‐of‐life is profound as patients suffer from chronic itching, respiratory distress, limiting dietary restrictions, social limitations, anxiety about accidental allergen exposure, and the psychological burden of unpredictable disease flares [99, 100, 101, 102, 103, 104].

Together, these factors underscore the urgent need for scalable, long‐lasting, and cost‐effective therapeutic strategies. With our current understanding, especially in industrialized nations, the pathological role of IgE in these diseases often outweighs any protective benefits mediated by the antibody.

### 1.5 Anti‐IgE Therapy

**FIGURE 3:**
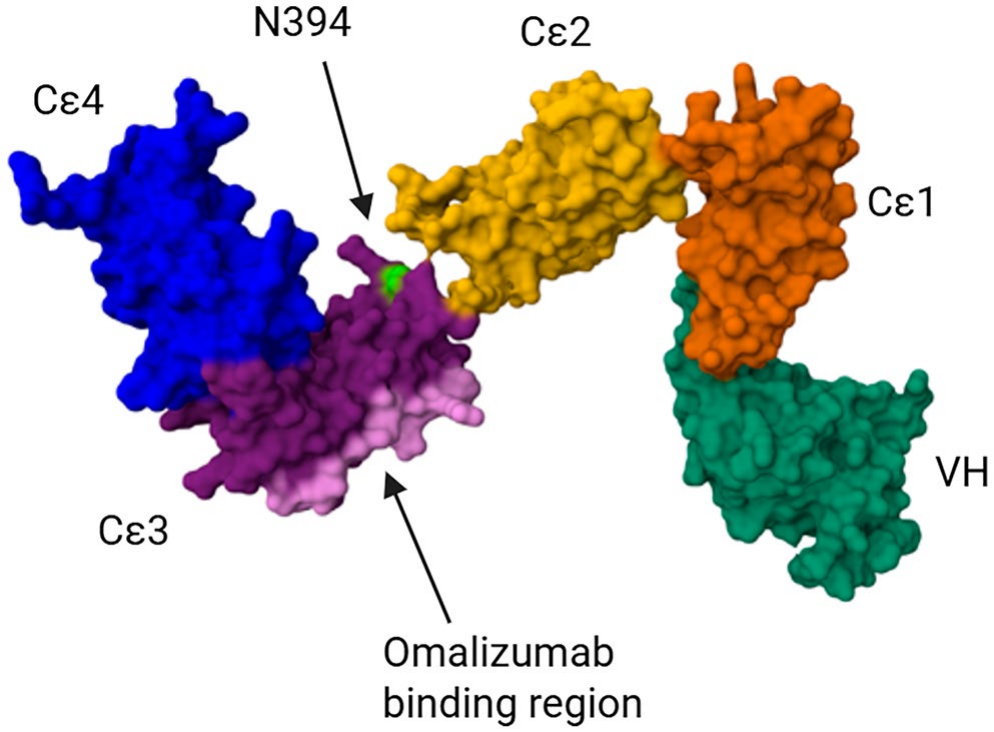
Anti‐IgE epitopes. Shown is the structure of human IgE (alphafold, AF‐P0DOX4‐F1) including the proposed omalizumab binding region (pink) located within the Cε3 domain (purple) [56]. Even if omalizumab does not bind directly to the N394 glycan, its recognition of IgE may structurally depend on the high mannose glycosylation site N394 (light green).

The therapeutic targeting of IgE has transformed the management of allergic diseases. Since its first approval by the FDA in 2003, the monoclonal anti‐IgE antibody omalizumab has become the prototype of anti‐IgE therapy and has been clinically evaluated in various indications (Table 1). It functions by binding to the Cε3 domain of IgE, preventing and disrupting its interaction with FcεRI on mast cells and basophils (Figure 3). This blocks both early‐ and late‐phase allergen‐induced reactions, reduces free IgE levels, and leads to downregulation of FcεRI expression on effector cells [56, 107, 123, 124, 125].

**TABLE 1:** Omalizumab indications.

Category	Indication	Population/criteria	References
Approved (global/strong regulatory consensus)	Severe allergic asthma	≥ 6 years; IgE‐mediated	[105, 106]
	Chronic spontaneous urticaria (CSU)	≥ 12 years; antihistamine‐refractory	[107, 108]
	Chronic rhinosinusitis with nasal polyps (CRSwNP)	Adults; inadequate response to intranasal steroids	[109, 110]
Approved (region‐specific)	Food allergy (e.g., U.S. FDA 2024 approval for reduction of allergic reactions)	Patients with IgE‐mediated food allergy; used with elimination diet	[111, 112]
Off‐label, possible benefits (case series, small trials, mechanistic rationale)	Allergic rhinitis	Allergic rhinitis uncontrolled with standard therapy	[113]
	Allergic bronchopulmonary aspergillosis (ABPA)	Asthma or cystic fibrosis with ABPA	[114]
	Aspirin‐exacerbated respiratory disease (AERD)	Patients with nasal polyps/asthma + aspirin sensitivity	[115]
	Atopic dermatitis (AD)	Moderate to severe AD	[116]
	Chronic inducible urticaria (CIU)	Antihistamine‐resistant inducible urticarias	[117]
	Idiopathic anaphylaxis	Recurrent episodes without clear trigger	[118]
	Mast cell disorders/mastocytosis	Symptoms from mast cell activation	[119]
	Drug allergy	Pretreatment to reduce risk of systemic reactions	[120]
	Eosinophilic esophagitis (EoE)	Select patient subsets	[121]
	Bullous pemphigoid (BP)	Refractory disease	[122]

Beyond severe allergic asthma, omalizumab is used for treatment of chronic spontaneous urticaria (CSU) and chronic rhinosinusitis with nasal polyps (CRSwNP) [107, 126]. The safety profile for omalizumab is overall excellent and a critical component to its success, though not all patients respond to the treatment [108, 127, 128, 129]. In CSU, higher total IgE levels are a positive indicator of therapy responsiveness [129, 130, 131]. Compared to various other monoclonals, omalizumab induces virtually no anti‐drug antibodies (ADA) [132]. A more recent use for omalizumab is in the treatment of food allergy or during allergy immunotherapy (AIT), where it may alter the maximally tolerated dose of allergen exposure [111, 112, 133, 134].

However, omalizumab requires regular administration, and remains very costly, limiting accessibility to the public [135]. Ligelizumab, a more novel next‐generation anti‐IgE antibody, was engineered with higher affinity for IgE and superior in vitro potency in blocking IgE–FcεRI interactions. In turn, ligelizumab has shown a reduced ability compared to omalizumab in neutralizing IgE:CD23 interaction [63]. Early clinical data, particularly in CSU, had suggested that ligelizumab might surpass omalizumab in efficacy, fueling expectations that higher affinity would translate into better clinical outcomes and lower injection doses across allergic diseases [136].

However, larger clinical trials have failed to demonstrate superiority of ligelizumab over omalizumab. Besides ligelizumab, other anti‐IgE biologics are being evaluated in preclinical and clinical studies, as reviewed by others [124, 137, 138, 139, 140]. Of note, ligelizumab appears to induce higher ADA responses than omalizumab, which were found in approximately 15% of the patients after 3–4 injections [137]. Overall, the case of ligelizumab raises questions about the mechanistic ceiling of IgE blockade. These findings are in line with the notion that, above a certain threshold, monoclonal antibody affinity may be irrelevant for neutralization [141].

Of note, the patent expiration of omalizumab (Xolair) now opens the market for a number of omalizumab biosimilars, the first of which (Omlyclo) has already been approved by the FDA [124, 142].

### 1.6 Natural Anti‐IgE Autoantibodies

A potential explanation for the safety of anti‐IgE therapy can be found in the physiological regulation of IgE levels. Natural anti‐IgE autoantibodies are present at surprisingly high levels in healthy individuals and may play an important physiological role in maintaining immune homeostasis by regulating circulating IgE levels [143, 144, 145].

Some have argued that dysregulated natural anti‐IgE could act pathogenically and favor anaphylaxis in certain disease contexts [144, 146, 147]. Possibly, natural anti‐IgE antibodies may also have detrimental effects by activating excessive cytokine secretion or by interfering with omalizumab therapy [131, 148]. Nevertheless, causal evidence and underlying mechanisms confirming the harmful nature of anti‐IgE responses in these contexts remain to be demonstrated.

Our own mechanistic studies in mice have rather suggested that natural anti‐IgE antibodies bind to free IgE, block FcεRI sensitization and facilitate IgE elimination in a similar fashion to omalizumab [149, 150]. These natural anti‐IgE antibodies could be transferred in passive immunization experiments to protect recipient mice from allergic anaphylaxis. Importantly, no anaphylactic side effects were observed in these experiments. Moreover, we have shown that the conserved mannose glycosylation site on IgE is critical for its self‐immunogenicity of IgE [149, 150]. Interestingly, omalizumab also depends on this conserved glycosylation site for human IgE recognition [151]. Thus, natural and therapeutic anti‐IgE antibodies compete with FcεRI for binding to IgE in a mutually exclusive manner, thereby preventing IgE from engaging FcεRI and triggering receptor activation. An interesting functional difference between natural and therapeutic anti‐IgE can be found in that IgE binding to CD23 is blocked by omalizumab, but seems to be actively enhanced with natural anti‐IgE antibodies [150, 151, 152, 153].

### 1.7 Is Active Vaccination Against IgE an Option?

Experience from respiratory syncytial virus (RSV) prevention provides a real‐world benchmark for comparing active vaccination with passive monoclonal antibody prophylaxis, with multiple economic evaluations of cost‐effectiveness. While long‐acting anti‐RSV monoclonals can offer strong short‐term protection mostly in babies, vaccines often emerge as the more scalable and economically favorable strategy [154, 155, 156].

Omalizumab continuous treatment costs have been estimated to range from $30,000–$60,000 per year, with injections every 2–4 weeks depending on the indication [157, 158, 159, 160]. These costs may limit omalizumab application for certain indications and make it less accessible to the broader public world‐wide. In contrast, a vaccination approach against IgE offers the potential for long‐lasting protection with a much lower financial burden, providing a cost‐effective alternative for patients and healthcare systems alike.

The development of IgE‐targeting vaccines has historically been hindered by concerns over mast cell and basophil activation through IgG recognition of receptor‐bound IgE. Yet, extensive evidence demonstrates that natural anti‐IgE antibodies do not activate effector cells. This physiological selectivity, likely regulated by the conserved mannose glycan, provides a robust mechanistic basis for safety. In other words, the existence and function of natural anti‐IgE autoantibodies demonstrate that the immune system is already capable of generating IgG anti‐IgE antibodies in a physiological and safe manner. Vaccination against IgE builds on this natural regulatory pathway, further supporting its safety and therapeutic potential in treating allergic diseases. Combined with the economic advantages and long‐term historical experience with active immunization, these insights establish a strong rationale for advancing anti‐IgE vaccination as a safe, effective, and sustainable therapeutic strategy for allergic diseases [105, 111, 129].

## 2 Anti‐IgE Vaccination Platforms

### 2.1 Adjuvanted IgE Fusion Proteins

The group of Lars Hellman has done pioneering work in designing vaccines against IgE. A first approach was based on engineered fusion proteins comprising the constant domains two (Cε2) and three (Cε3) of rat IgE, fused to either the glutathione‐S‐transferase (GST) protein from *Schistosoma japonicum* or the maltose binding protein (MBP) from *Escherichia coli* in order to increase stability and T helper responses. These fusion proteins elicited strong and safe anti‐IgE autoimmune responses in rats. Up ovalbumin‐sensitized rats, vaccination with the GST‐C2C3 fusion protein led to a marked reduction in serum IgE levels and, subsequently, inhibition of mast cell and basophil degranulation. This demonstrated that anti‐IgE vaccines can reduce IgE levels sufficiently to produce therapeutic effects [161, 162, 163].

The group next designed vaccines based on chimeric IgE molecules consisting of Cε2, Cε3, and Cε4 domains derived from two different species [164]. The FcεRI‐binding domain, Cε3, originated from rats, the experimental target species, while the flanking Cε2 and Cε4 domains were sourced from the evolutionarily distant mammal opossum. These foreign domains were intended to structurally support the Cε3 region and provide nonself T cell epitopes to overcome immune tolerance and stimulate a robust antibody response. In addition, the concept was to focus protective antibody responses on the rat Cε3‐IgE domain to avoid IgE cross‐linking on IgE bound to mast cells. The efficacy of this approach was again evaluated in actively sensitized rats. Vaccination induced anti‐IgE antibodies in all animals and led to a substantial reduction in serum IgE levels. Notably, vaccinated rats displayed significantly reduced skin reactivity following allergen challenge. The vaccine was well tolerated, with no evidence of IgE cross‐linking activity in sera from vaccinated animals. Moreover, the immune response waned over time, suggesting reversibility. However, the adjuvant system used for these fusion proteins in rats needed to be harsh, using complete Freunds Adjuvant (CFA) and boosters with Incomplete Freunds adjuvant (IFA).

A similar approach was then used in dogs where allergic diseases are common, yet current treatments often fail to provide sufficient relief. Here, the chimeric vaccine contained dog Cε3 flanked by opossum Cε2 and Cε4 regions. Anti‐IgE vaccines formulated with oil‐in‐water (O/W) adjuvants successfully induced high anti‐IgE antibody levels in Beagle dogs and reduced total IgE by an average of 65%, suggesting promising potential for treating atopic diseases in dogs [165].

### 2.2 Adjuvanted Synthetic IgE Peptide Approaches

Other groups designed vaccines based on synthetic IgE peptides, with the same idea of preventing IgE binding to FcεRI through active immunization. In contrast to protein vaccines, peptides can be synthesized without the need for recombinant expression systems, greatly facilitating good manufacturing practices (GMP)–based production, the rate limiting step in today's biologics development. However, it is often difficult to generate strong and specific antibody responses with peptides because target epitopes are often conformational. Thus, peptide vaccines often rely on modifications and carrier systems for improved immunogenicity and often induce antibodies of relatively low affinity [166, 167].

A peptide derived from the conserved positions 413–435 of the Cε3 loop region, modified to enhance conformational stability, elicited anti‐IgE antibodies that effectively blocked IgE‐mediated histamine release. To enhance immunogenicity, the peptide was linked to a T‐helper epitope, creating a fully synthetic immunogen and formulated in CFA. This vaccine induced polyclonal, site‐specific anti‐IgE antibodies that blocked histamine release from human IgE‐sensitized basophils and reduced passive cutaneous anaphylaxis in rats. In dogs, vaccination with O/W adjuvant led to significant induction of anti‐IgE responses and reductions in total serum IgE levels, demonstrating the potential of this vaccine to safely modulate IgE‐mediated allergic responses [168].

Virus‐like Particles (VLPs) are vaccine carrier platforms inspired by the self‐assembling structural proteins of viruses, which can form noninfectious particles that mimic viral size and geometry and thus strongly enhance innate and adaptive immune responses. A peptide‐based VLP‐IgE vaccine was developed by using human IgE receptor‐binding site peptides conjugated to hepatitis B surface antigen, formulated with an adjuvant system consisting of liposomes and CpG oligonucleotides. In rats, the vaccine induced high‐titer IgG antibodies that recognized free but not receptor‐bound IgE and blocked IgE‐receptor interaction. In both preventive and therapeutic models, vaccinated mice and rats showed significantly reduced allergen‐specific IgE levels compared to controls. These findings demonstrate that IgE peptide‐based vaccines can safely induce anti‐IgE antibodies and effectively reduce IgE‐mediated allergic responses [169].

A similar vaccine was developed by Pfizer, based on bacteriophage Qβ VLP linked to Cε3 peptides in collaboration with our group. These VLPs are self‐adjuvanted through their enclosed bacterial RNA that activates TLR7/8 [170]. In mice and nonhuman primates, this vaccine was able to reduce IgE levels. However, in nonhuman primates, the anti‐IgE vaccine did not work consistently in all subjects. In a Phase I clinical trial including allergic rhinitis patients, the vaccine was safe and immunogenic in a dose‐dependent manner, but it only had a moderate effect on the downregulation of IgE levels [170, 171, 172, 173].

The reasons for the lack of efficacy of these peptide vaccines will need to be further understood and studied in future clinical trials. As mentioned, despite their simplicity in terms of production, peptide vaccines often fail to generate high‐affinity antibodies with neutralizing abilities. Most likely, the problem could be solved by using whole IgE domains as opposed to only peptides to induce antibodies of higher affinity, and/or by targeting conformational epitopes, which have also shown more promise in preclinical models.

### 2.3 IgE Domains Displayed on Immunogenic Recombinant Carriers

**FIGURE 4:**
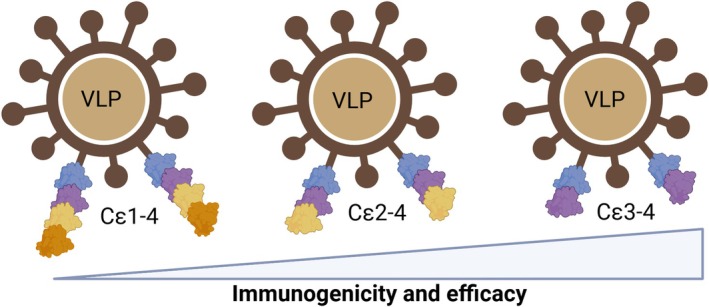
VLP vaccine candidates (Cε1‐4, Cε2‐4, and Cε3‐4). Schematic representation and comparative analysis of the three IgE‐derived vaccine constructs Cε1‐4, Cε2‐4, and Cε3‐4. Shortening of the IgE construct was associated with increased immunogenicity and superior in vivo efficacy.

Recently, our group has developed such vaccines, based on self‐adjuvanted cucumber mosaic virus (CuMV) derived VLP vaccines consisting of different recombinant IgE Fc fragments that were chemically coupled to the VLP carrier. In mice, these candidates generated robust and long‐lasting anti‐IgE responses, reduced free and cell‐bound IgE levels, and protected mice against allergen challenges in models of local and systemic allergic responses. Importantly, consistent with findings from other groups, anti‐IgE vaccinated allergic mice showed no signs of adverse allergic reactions. Three different IgE Fc fragment lengths, Cε1‐Cε4, Cε2‐Cε4, Cε3‐Cε4 were tested (Figure 4). While all fragments showed immunogenicity, Cε1‐Cε4 was the least immunogenic and protective, whereas the shortest fragment Cε3‐Cε4 performed best in terms of immunogenicity and protection [174]. In a follow‐up study, we further investigated the mechanism of action of the most promising candidate VLP‐Cε3‐Cε4 more closely, showing that the protection can occur independently of the regulatory Fc receptors FcγRIIb and FcεRII (CD23) [59, 175].

**FIGURE 5:**
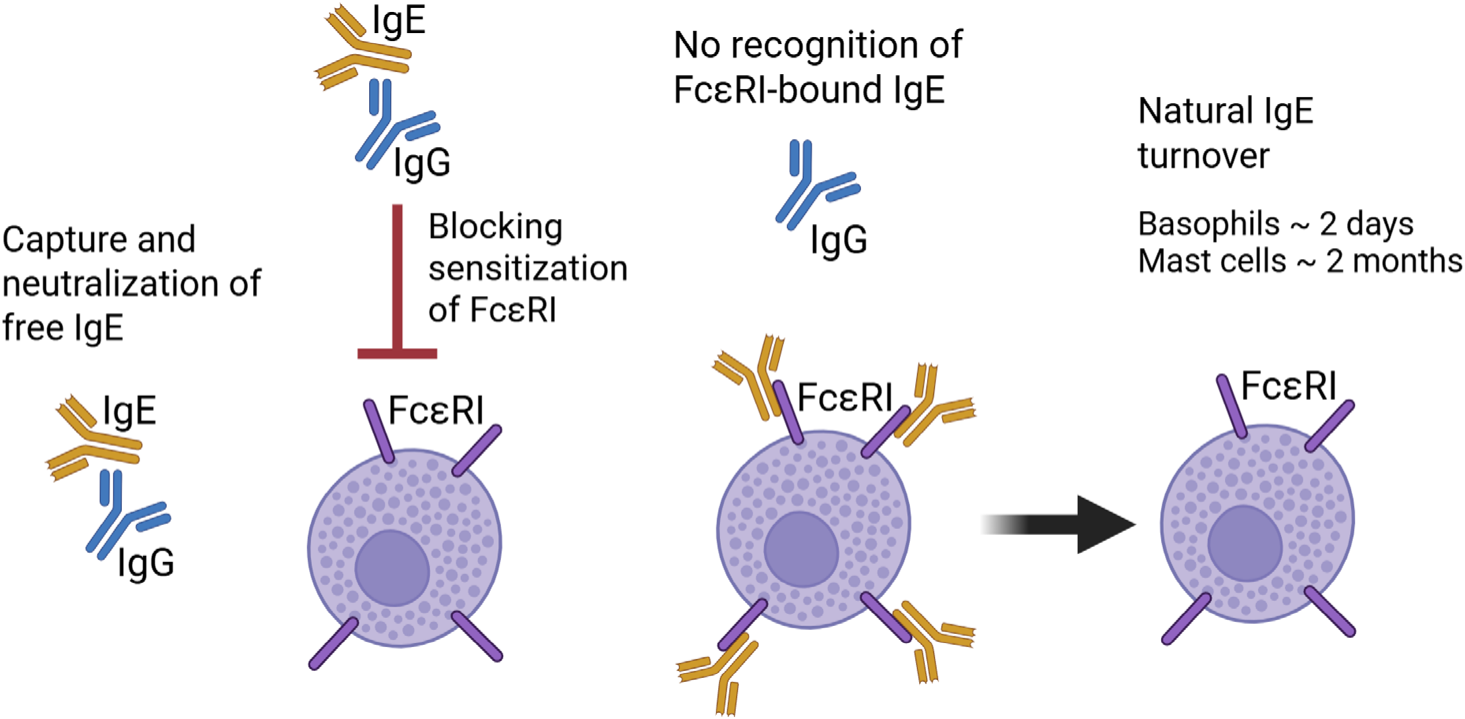
Mechanisms of vaccine‐induced anti‐IgE antibody action on free and receptor‐bound IgE. Anti‐IgE antibodies capture and neutralize free IgE, preventing sensitization of FcεRI on effector cells. Because they do not recognize FcεRI‐bound IgE, effector cells gradually lose surface IgE through natural receptor turnover—rapid in the more short‐lived basophils (~2 days) and more slowly in mast cells (~2 months).

We then further characterized the antibodies induced by the anti‐IgE vaccine in vitro in bone marrow‐derived mouse mast cells (BMMCs). Interestingly, the vaccine‐induced antibodies prevented IgE binding to FcεRI but did not recognize and bind IgE that was already displayed on FcεRI. In line with these in vitro findings, vaccine‐induced anti‐IgE antibodies did not trigger anaphylaxis upon adoptive transfer into allergic mice. These experiments could explain the excellent preclinical safety of all the different anti‐IgE vaccine candidates. In line with our previous findings, we observe that the recognition of IgE by vaccine‐induced anti‐IgE antibodies depends on the conserved high‐mannose on IgE for binding; the same glycosylation that is involved in IgE binding to FcεRI as well as IgE recognition by omalizumab and natural anti‐IgE antibodies [79, 176] (Figure 5).

In a similar approach by Conde et al., human IgE Cε3–Cε4 fragment was coupled on the nontoxic mutant diphtheria toxin cross‐reactive material 197 (CRM197) as an immunogenic vaccine platform, formulated with a squalene‐based O/W adjuvant [177]. CRM197 is a nontoxic mutant of diphtheria toxin developed as a protein carrier to enhance antigen immunogenicity by recruiting T‐cell help.

The human fragment was designed with a G335C mutation that locks IgE in its “closed” conformation, designed to prevent FcεRI activation. Vaccine efficacy was then evaluated in a mouse strain humanized for both IgE and the high‐affinity IgE receptor FcεRI. Immunization of allergic mice elicited sustained production of neutralizing anti‐human IgE antibodies for up to 12 months without detectable adverse effects and conferred robust protection against anaphylaxis. In line with our previous findings, the recognition of IgE by anti‐IgE antibodies was likewise found to be N394‐dependent. Finally, this study also elegantly showed that anti‐IgE vaccination does not alter the immune response to parasites, which is in line with the currently available data on the safety of omalizumab in that regard [178].

## 3 Conclusions and Perspectives

Extensive preclinical research with various platforms and even a phase I trial in humans has shown that vaccination against IgE is feasible and safe, with no evidence of mast cell degranulation or systemic anaphylaxis.

Based on current preclinical studies, we hypothesize that the more recently developed full Cε3–Cε4 domain vaccine candidates will outperform the adjuvanted protein or peptide‐based approaches in the clinics due to their improved ability to induce antibodies with neutralizing abilities (Table 2).

**TABLE 2:** Anti‐IgE vaccine platforms.

Anti‐IgE platform	Advantages	Disadvantages	References
Protein + adjuvant	Increased antibody quality	Weak immunogenicity, often needs strong adjuvant/delivery	[164, 165]
Peptide + adjuvant	Very simple production	Weak immunogenicity; reduced antibody quality; often needs strong adjuvant/delivery	[168, 169]
Peptide–VLP (self‐adjuvanted)	Relatively simple production, strong immunogenicity, reduced need for adjuvant	Reduced antibody quality; anti‐carrier immunity possible	[170, 173]
Protein–CRM197 conjugate + adjuvant	Increased antibody quality, carrier boosts immunogenicity	Typically still needs adjuvant/boosts; anti‐carrier immunity possible	[177]
Protein–VLP (self‐adjuvanted)	Increased antibody quality, strong immunogenicity; reduced need for adjuvant	Anti‐carrier immunity possible	[174, 176]

Self‐adjuvanted VLPs have the general advantage of eliciting the most potent humoral responses, even over CRM carriers which often require additional adjuvantation. In turn, anti‐carrier responses also occur, which could alter immune responses as observed for both CRM and VLP‐based vaccine platforms in a comparative study [179]. Importantly, the impact of preexisting anti‐carrier immunity is difficult to dissect experimentally, as prior exposure can also induce immune imprinting (“original antigenic sin”), favoring recall of carrier‐specific memory responses and confounding interpretation of antibody‐mediated effects. Increasing the Cε3–Cε4 antigen density on CRM/VLP systems may reduce anti‐carrier responses. Importantly, in preclinical models, preexisting anti‐carrier antibodies do not prevent efficient boost‐responses to VLP vaccines [174, 180].

Regading the safety of vaccination, existing literature challenges widely held concerns that targeting IgE with vaccines might provoke harmful immune activation. We are not aware of preclinical in vivo evidence showing that harmful anti‐IgE responses are generated by anti‐IgE vaccination. In contrast, anti‐IgE antibodies have been safely induced in mice, rats, dogs, nonhuman primates, and even in a phase I clinical trial in humans. Clinical experience with omalizumab suggests that anti‐parasite or anti‐cancer immune responses are not impeded with anti‐IgE therapy, but obviously, this will need to be confirmed for the vaccine candidates. Reevaluating safety concerns in clinical trials is essential to unlocking the full therapeutic potential of this approach.

In addition to making current anti‐IgE treatment indications in asthma, CSU, CRSwNP, and AIT more feasible world‐wide, the reduced costs of anti‐IgE vaccination could unlock new indications for anti‐IgE therapy that are currently limited by the cost‐effectiveness of omalizumab. The theoretical reduction of injections needed with an anti‐IgE vaccine could also be beneficial in terms of patient quality‐of‐life or treatment adherence [181]. Whether omalizumab partial or nonresponders could benefit from anti‐IgE vaccination is another question to be addressed in the future. Projecting further on the drastic global rise of atopic diseases world‐wide, with allergic sensitization reaching almost 50% in some areas, anti‐IgE vaccination could be envisioned as a preventive approach already in children.

In summary, anti‐IgE vaccination could be a promising strategy for managing allergic diseases that aligns with natural IgE immune regulation.

## Funding

This work was supported by Schweizerischer Nationalfonds zur Förderung der Wissenschaftlichen Forschung, 228774 (PE), 10001271 (MFB/PE), 185114 (MFB/MV).

## Conflicts of Interest

Martin F. Bachmann is involved and holds shares in companies related to VLP‐based immunotherapies. The other authors declare no relevant conflicts of interest.

## Data Availability

The authors have nothing to report.
